# Deep learning-assisted widefield endothelial imaging in Descemet membrane endothelial keratoplasty

**DOI:** 10.3389/fmed.2025.1715673

**Published:** 2025-11-18

**Authors:** Ezekiel Ze Ken Cheong, Khin Yadanar Win, Qiu Ying Wong, Ralene Sim, Clarissa Ng Yin Ling, Hla Myint Htoon, Hon Shing Ong, Jodhbir S. Mehta, Bingyao Tan, Leopold Schmetterer, Marcus Ang

**Affiliations:** 1 Ophthalmology and Visual Sciences Academic Clinical Programme, Duke-NUS Medical School, Singapore, Singapore; 2 Singapore National Eye Centre, Singapore, Singapore; 3 Singapore Eye Research Institute, Singapore, Singapore; 4 SERI-NTU Advanced Ocular Engineering (STANCE) Program, Singapore, Singapore; 5 Centre for Medical Physics and Biomedical Engineering, Medical University of Vienna, Vienna, Austria

**Keywords:** Descemet membrane endothelial keratoplasty, corneal endothelium, widefield specular microscopy, deep learning, image quality, endothelial cell density, endothelial cell loss

## Abstract

**Purpose:**

Significant endothelial cell density (ECD) losses in Descemet membrane endothelial keratoplasty (DMEK) can precipitate graft failure. Currently, clinical practice only evaluates the central corneal endothelium and not the paracentral or peripheral cornea. Here, we evaluate a deep learning (DL) algorithm for automated image quality assessment and detection of low corneal ECD in widefield specular microscopy (WFSM) images following DMEK, at central, paracentral and peripheral regions.

**Methods:**

Prospective observational study in eyes with clear, stable DMEKs. WFSM imaging (CEM-530; Nidek, Japan) performed at a baseline visit (mean of 23 months post-DMEK) and 6 months after. Images at 15 locations were captured; 1 central, 8 paracentral, and 6 peripheral. Image quality and corneal endothelial cell parameters including ECD were assessed. SqueezeNet architecture was employed for binary and multi-class automated image quality and low ECD detection (threshold set at < 1,000 cells/mm^2^). Main outcome measures were regional ECD and area under curve (AUC) for DL tasks.

**Results:**

DMEK was performed in 53 eyes (43 subjects, mean age 67.4 ± 10.1 years) with Fuchs endothelial corneal dystrophy (FECD) (71.7%; 38 eyes) and pseudophakic bullous keratopathy (PBK) (28.3%; 15 eyes). The DL classification models were trained and validated on 1,362 images, achieving AUCs of 0.979 (binary image quality), 0.907 (multi-class image quality) and 0.980 (low ECD detection). WFSM imaging was able to detect both central ECD loss (1,709 vs. 1,555 cells/mm^2^; *p* < 0.001) and peripheral ECD loss (1,498 vs. 1,347 cells/mm^2^; *p* = 0.016). Overall, we estimated a mean annual central ECD loss of 5.81% (95% CIs: 3.54–8.08%) in the stable phase of our DMEK cohort.

**Conclusion:**

Serial WFSM was able to image a larger area of the endothelium in eyes with DMEK. Our DL model was a useful adjunct that analysed the large number of specular microscopy images to identify scans of adequate quality and ECD based on pre-defined thresholds. This approach may support the use of DL-assisted WFSM for early detection of ECD loss which may aid in monitoring endothelial health and graft survival following DMEK.

## Introduction

Descemet membrane endothelial keratoplasty (DMEK) has gained popularity over Descemet stripping automated endothelial keratoplasty (DSAEK) for the treatment of endothelial failure, as it can achieve better visual outcomes (1–3), quicker recovery (4), with lower rejection rates (5, 6). However, endothelial cell loss (ECL) is an inevitable consequence of any successful endothelial keratoplasty (7, 8). Even after a successful DMEK, endothelial cell density (ECD) continues to decline over time, with the rate of decline reaching a plateau around 12 months post-operatively (9). Excessive post-operative ECL can lead to late graft failure, which has been reported in up to 30% of patients within 5 years (10, 11). Factors such as glaucoma (12, 13), rejection (14) and uveitis (15) contribute to long-term ECL, highlighting the importance of post-operative monitoring of the cornea endothelium and ECD to ensure long-term graft survival.

Post-operative monitoring of ECD is typically performed in a limited, central region (0.55 mm x 0.25 mm) of the entire corneal endothelial area using specular microscopy (16–20). This proxy does not fully reflect the overall endothelial health, as it only represents a thousandth of the entire endothelial surface area (21). Evaluating the entire graft, including the paracentral and peripheral regions, is important to understanding cell migration and morphology associated with ECL, which can differ significantly between conditions such as Fuchs endothelial cell dystrophy (FECD) and pseudophakic bullous keratopathy (PBK) (22). Accurate ECD measurement depends on high-quality imaging, which is often compromised in corneal edema. Moreover, as imaging volumes increase, substantial time is required to validate the reliability of automated cell segmentation, posing a challenge for consistent and reproducible analysis.

To address these challenges, we developed a deep learning (DL) framework consisting of three independently trained models fine-tuned from a pre-trained SqueezeNet architecture (23), for automated binary and multi-class imaging grading, as well as low ECD detection. Binary image quality assessment, for the selection of reliable images, is necessary prior to low ECD detection. To our knowledge, no comprehensive studies have characterized post-DMEK corneal endothelial cell (CEC) changes in the paracentral and peripheral regions using widefield specular microscopy (WFSM), particularly those employing DL techniques. Therefore, this study aims to evaluate longitudinal changes in ECD over a 6-month period, at the central and peripheral cornea following DMEK, using WFSM integrated with DL.

## Materials and methods

### Study design and participants

We conducted a longitudinal study with patients recruited between January 2022 and December 2022 from a tertiary ophthalmology centre - Singapore National Eye Centre. We included adults aged 21 years or older, who had undergone DMEK for corneal decompensation due to FECD or PBK as surgical indications. All patients had DMEK for at least one year to ensure cornea clarity and stable endothelial stability (defined as unchanged central ECD for past 2 visits). A WFSM scan was done at a baseline visit, and repeated 6 months later. Basic demographic data, glaucoma status and indications for DMEK were obtained and compiled from electronic health records, along with DMEK donor graft details such as age, gender, graft ECD, coefficient of variation (CV) and hexagonality (HEX). Our study was conducted in accordance with the Declaration of Helsinki, with informed consent obtained from all subjects and ethics approval granted by the SingHealth Centralized Institutional Review Board (CIRB R1501/84/2017).

### Clinical assessment

All patients were clinically evaluated by three cornea specialists (MA, JM, HSO). Best-corrected visual acuity (BCVA) was measured using Snellen chart and subsequently converted to logarithm of minimum angle of resolution (logMAR) units for statistical analysis (24). Finger counting, hand movement perception, light perception and no light perception were graded as 1.9, 2.3, 2.7 and 3.0 logMAR, respectively (25–27). All investigations and clinical evaluation were conducted at a baseline visit and repeated at a 6-month follow-up as per the Singapore Corneal Transplant Registry protocol (28).

### Regional analyses using widefield specular microscopy

**Figure 1:**
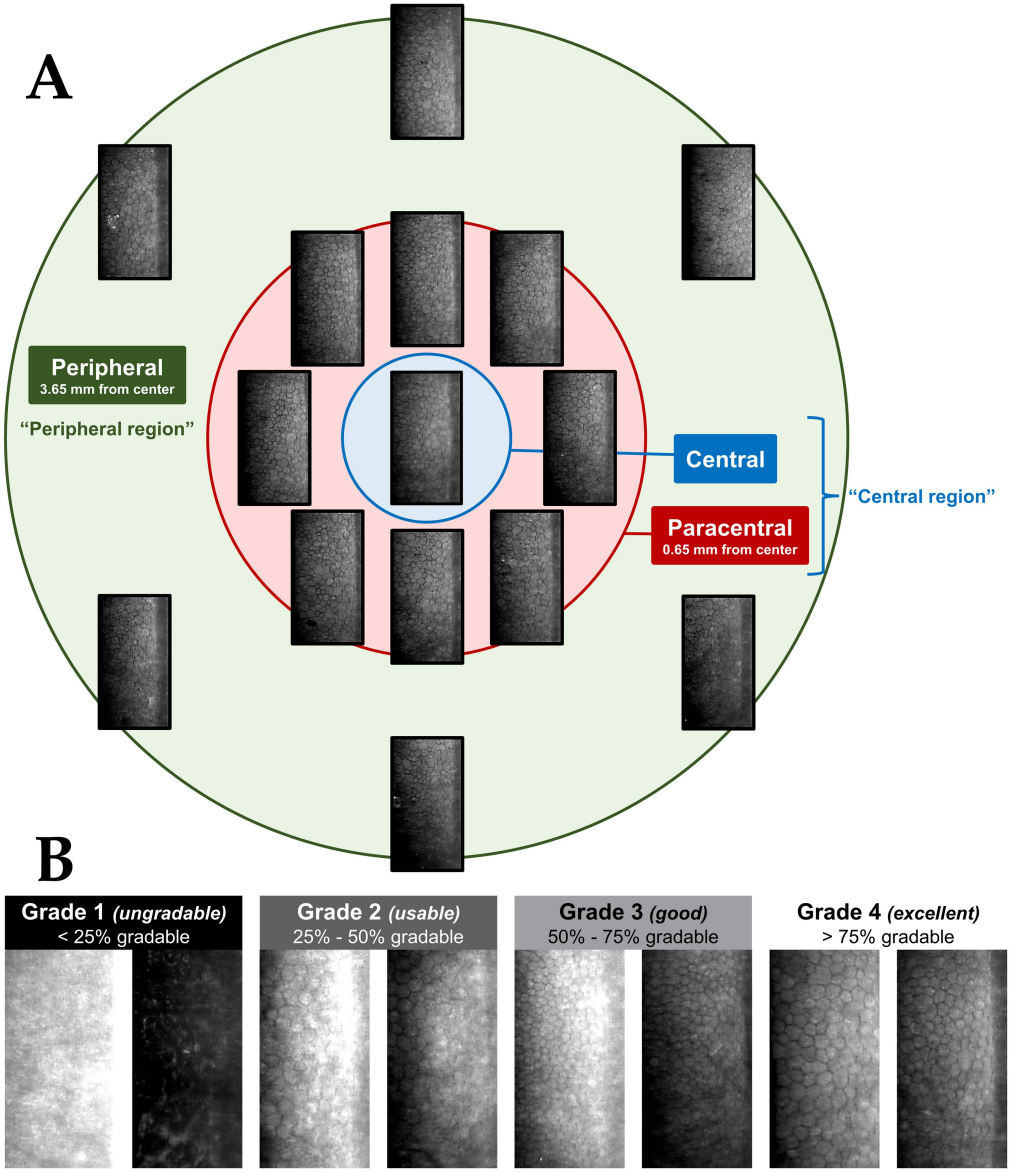
**(A)** Example of a widefield specular microscopy (WFSM) scan from the Nidek CEM-530. The different locations are depicted as: blue for central, red for paracentral and green for peripheral. Each scan consists of 1 central, 8 paracentral and 6 peripheral images of the corneal endothelium. The central and paracentral images comprise the central region within a 1.3 mm diameter. The peripheral region is an outer annulus that is 7.3 mm in diameter. **(B)** Examples of specular images with various quality grades (1–4). Grade 1 images (ungradable) had less than 25% of the total area being gradable. Grade 2 images (usable) had 25%–50%, grade 3 images (good) had 50%–75% and grade 4 images (excellent) had more than 75% of the total area being gradable. This grading scheme was adapted from Huang et al. (29).

All scans were performed using a widefield specular microscopy technique (CEM-530; Nidek, Japan). As illustrated in Figure 1A, each scan achieves an image in 15 corneal locations: 1 central, 8 paracentral and 6 peripheral images. Paracentral images are 0.65–0.80 mm from the centre and peripheral images are 3.65–4.00 mm from the centre. In our analysis, the central and paracentral images were considered the “central region” of the cornea, with the peripheral images comprising the “peripheral region”. The area of each captured image was 0.55 mm by 0.25 mm. For all eyes, image quality, ECD, corneal thickness (CT), CV and HEX obtained from the built-in software were compiled and averaged within their respective regions; central or peripheral. These parameters, except for image quality, were excluded from further analyses if more than half of the images in that region (ie. ≥ 5 central, ≥ 4 peripheral) had image quality graded as 1 (ungradable) by any of the two independent graders. Image quality grading is described in the following section.

### Image quality grading

The image quality of each image was graded by two trained graders (EC, QW) independently based on a standardized grading scheme adapted from Huang et al. (29) The modified scheme is based on the estimated percentage of the total image area that can be confidently graded as depicted in Figure 1B. For further analysis of image quality distribution as well as group classification for DL, the lower of two image quality grades was selected in disagreements, in order to be more conservative.

### Deep learning architecture

The DL frameworks were trained separately for three main tasks: binary image quality grading [usable (grade ≥ 2) vs. ungradable (grade 1)], multi-class image quality grading (grades 1 to 4) and ECD classification (< 1,000 cells/mm^2^ vs. ≥ 1,000 cells/mm^2^). For all three tasks, we employed the same DL architecture based on transfer learning with a pre-trained SqueezeNet model (23). Specifically, SqueezeNet was selected after preliminary comparisons with other convolutional neural networks (including ResNet-50, DenseNet-201, EfficientNet-B0, and ShuffleNet). SqueezeNet achieved comparable or superior performance to larger networks while being lightweight (~1.2 M parameters vs. ~25 M for ResNet-50, ~20 M for DenseNet-201, ~5.3 M for EfficientNet-B0, and ~2.3 M for ShuffleNet) and computationally efficient, which is advantageous for resource-limited clinical applications. In preliminary experiments on image quality grading, SqueezeNet achieved performance comparable or superior to other tested architectures, including ResNet-50, DenseNet-201, EfficientNet-B0 and ShuffleNet. Given its efficiency and smaller parameter size, SqueezeNet was adopted as the final model for all classification tasks. The same transfer learning framework and hyperparameter settings were applied across all architectures to ensure a fair comparison.

**Figure 2:**
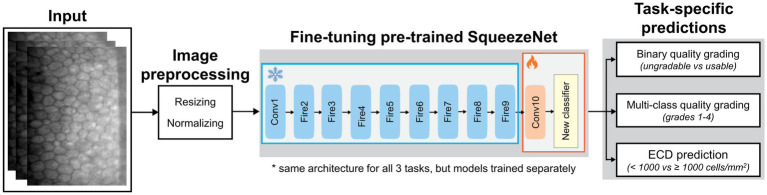
Deep learning pipeline for automated image quality grading and ECD prediction using a fine-tuned SqueezeNet1. The early layers are frozen to retain ImageNet-learned features, while only the final convolutional and classifier layers are fine-tuned using specular microscopy images. The same architecture is used across the three tasks.

As illustrated in Figure 2, the initial convolutional and Fire modules (Conv1 to Fire9) were frozen to retain general visual features learned from ImageNet, while the final layers (Conv10 and the classifier) were fine-tuned using task-specific datasets of specular microscopy images. This transfer learning technique offers efficient adaptation to limited labelled medical data while preventing overfitting. Each model was trained independently on its respective task. During training, all images were resized to 300 × 200 pixels and normalized using ImageNet mean and standard deviation. We used a batch size of 32 and trained the models using the Adam optimizer with a learning rate of 0.0005, l2 regularization of 0.0005 and cross-entropy loss. Early stopping and learning rate scheduling were applied based on validation performance. Only the final convolutional layer and classifier were updated during training; all earlier layers were frozen to retain the pre-trained weights. For model interpretability, we used gradient-weighted class activation mapping (Grad-CAM) heatmaps to visualize the key regions that influence model’s classification of certain grades. Grad-CAM generates class-specific heatmaps by using gradients from the target output flowing into the final convolutional layer (30).

### Deep learning data partitioning

Firstly, the binary image quality grading task was designed as a pre-screening step to automatically identify and exclude ungradable images from downstream analysis. Images were grouped as ungradable (grade 1) or usable (grade ≥ 2). The dataset consisted of a total of 1,362 images, of which 18.9% (*n* = 258) were ungradable and 81.1% (*n* = 1,104) were usable. This dataset of 1,362 images were then split into 80% training and validation and 20% for testing. Secondly, multi-class image quality grading was designed to categorize images into four quality levels (grades 1–4) to reflect the severity of quality degradation. The data was stratified by image quality grade and anatomical regions (central, paracentral and peripheral) to ensure balanced representation across dataset partitions. Lastly, for ECD classification, with input quality ensured by excluding images graded as grade 1 (ungradable) from the dataset, the remaining images were dichotomized into two ECD classes: < 1,000 and ≥ 1,000 cells/mm^2^. Thus, the final dataset included 1,104 images and was partitioned into training, validation, and test sets.

### Statistical analyses

SPSS 26.0 (SPSS; IBM Corp, USA) and GraphPad Prism software (Prism; GraphPad, USA) were used for all statistical analyses in this study. All between-groups comparisons of continuous parameters were performed using independent t-tests (paired tests for applicable comparisons). All between-groups comparisons of categorical parameters were performed using Fisher’s exact or chi-square tests. For multivariable linear regression, variables were chosen by an automatic linear modelling process in SPSS 26.0, using forward stepwise regression. Upper and lower 95% confidence intervals (CIs) were used for graphs and means. Tests were two-sided with statistical significance set at *p* < 0.05. Categorical variables are detailed as number (%), and continuous variables are detailed as mean ± SD. Asterisks and bold *p*-values indicate statistical significance. DL model performance was evaluated using accuracy, precision, recall, F1 score, and area under curve (AUC). Mean annual ECD loss was estimated using a scatter plot of percentage of donor ECD remaining over time post-DMEK. The percentage of donor ECD remaining was defined as the central ECD obtained from WFSM over the pre-operative DMEK donor graft ECD.

## Results

### Demographics

We included 53 eyes with surviving DMEK grafts (43 subjects) at least 1 year after surgery (mean 23.2 ± 20.6 months) that demonstrated clinically stable cornea clarity and stable ECD. Demographics and clinical summary are detailed in Table 1. Mean age of our subjects (at the time of DMEK) was 67.4 ± 10.1 years, 71.7% of the eyes had DMEK for cornea decompensation due to FECD (*n* = 38) and 28.3% for PBK (*n* = 15). Of the 53 eyes, 35.8% had pre-existing glaucoma (*n* = 19) and 64.2% did not have glaucoma (*n* = 34).

**Table 1:** Descriptive characteristics of the subject population at first baseline visit, along with DMEK donor graft characteristics.

Characteristics	All eyes *n* = 53	Glaucoma status		*P*
		Glaucomatous *n* = 19	No glaucoma *n* = 34	
DMEK recipient characteristics				
Age at 1st visit (years)	69.4 ± 10.0	70.5 ± 10.6	68.7 ± 9.8	0.543
Time since DMEK (months)	23.2 ± 20.6	20.3 ± 20.1	24.9 ± 21.0	0.448
Age at DMEK (years)	67.4 ± 10.1	68.8 ± 10.1	66.7 ± 10.2	0.463
Ethnicity				
Chinese	46 (86.8%)	16 (84.2%)	30 (88.2%)	0.868
Indian	3 (5.7%)	2 (10.5%)	1 (2.9%)	
Malay	3 (5.7%)	1 (5.3%)	2 (5.9%)	
Others	1 (1.9%)	0	1 (2.9%)	
Gender				
Male	27 (50.9%)	14 (73.7%)	13 (38.2%)	0.021*
Female	26 (49.1%)	5 (26.3%)	21 (61.8%)	
BCVA (logMAR)	0.30 ± 0.29	0.33 ± 0.18	0.29 ± 0.33	0.628
Indication for DMEK				
FECD	38 (71.7%)	9 (47.4%)	29 (85.3%)	<0.01**
PBK	15 (28.3%)	10 (52.6%)	5 (14.7%)	
DMEK donor characteristics				
Age (years)	61.9 ± 7.0	62.7 ± 7.6	61.5 ± 6.8	0.574
Gender				
Male	35 (66.0%)	13 (68.4%)	22 (64.7%)	1.00
Female	18 (34.0%)	6 (31.6%)	12 (35.3%)	
ECD (cells/mm^2^)	2,849 ± 228	2,791 ± 187	2,881 ± 246	0.174
CV (%)	34.1 ± 3.2	34.0 ± 2.4	34.1 ± 3.5	0.923
HEX (%)	56.4 ± 6.1	57.1 ± 4.9	55.9 ± 6.7	0.513

*Bolded *p*-values with asterisks indicate statistical significance. BCVA, best corrected visual acuity; FECD, Fuchs endothelial cell dystrophy; PBK, pseudophakic bullous keratopathy; ECD, endothelial cell density; CV, coefficient of variation; HEX, hexagonality.

### Quality of widefield specular microscopy images

Image quality of all WFSM images across 2 visits (*n* = 1,590) was generally good, where graders had 87.0% agreement. All disagreements were within 1 grade disparity. 83% (795/954) of central and paracentral images were at least grade ≥ 2 and 65% (622/954) were at least grade ≥ 3. Peripheral scans had poorer image quality scores compared to central and paracentral scans (both *p* < 0.0001). 53% (334/636) of peripheral images were at least grade ≥ 2 and 25% (161/636) were at least grade ≥ 3.

### Changes in widefield specular microscopy parameters

The mean image quality in the central region did not significantly change over 6 months (2.79 vs. 2.80; *p* = 0.926) but there was a significant decline in peripheral region image quality (2.09 vs. 1.72; *p* < 0.001). ECD declined significantly at the central (1,709 vs. 1,555 cells/mm^2^; *p* < 0.001) and peripheral regions (1,498 vs. 1,347 cells/mm^2^; *p* = 0.016). There were no significant changes observed in CV, HEX, and CT.

### Endothelial cell density decline

**Figure 3:**
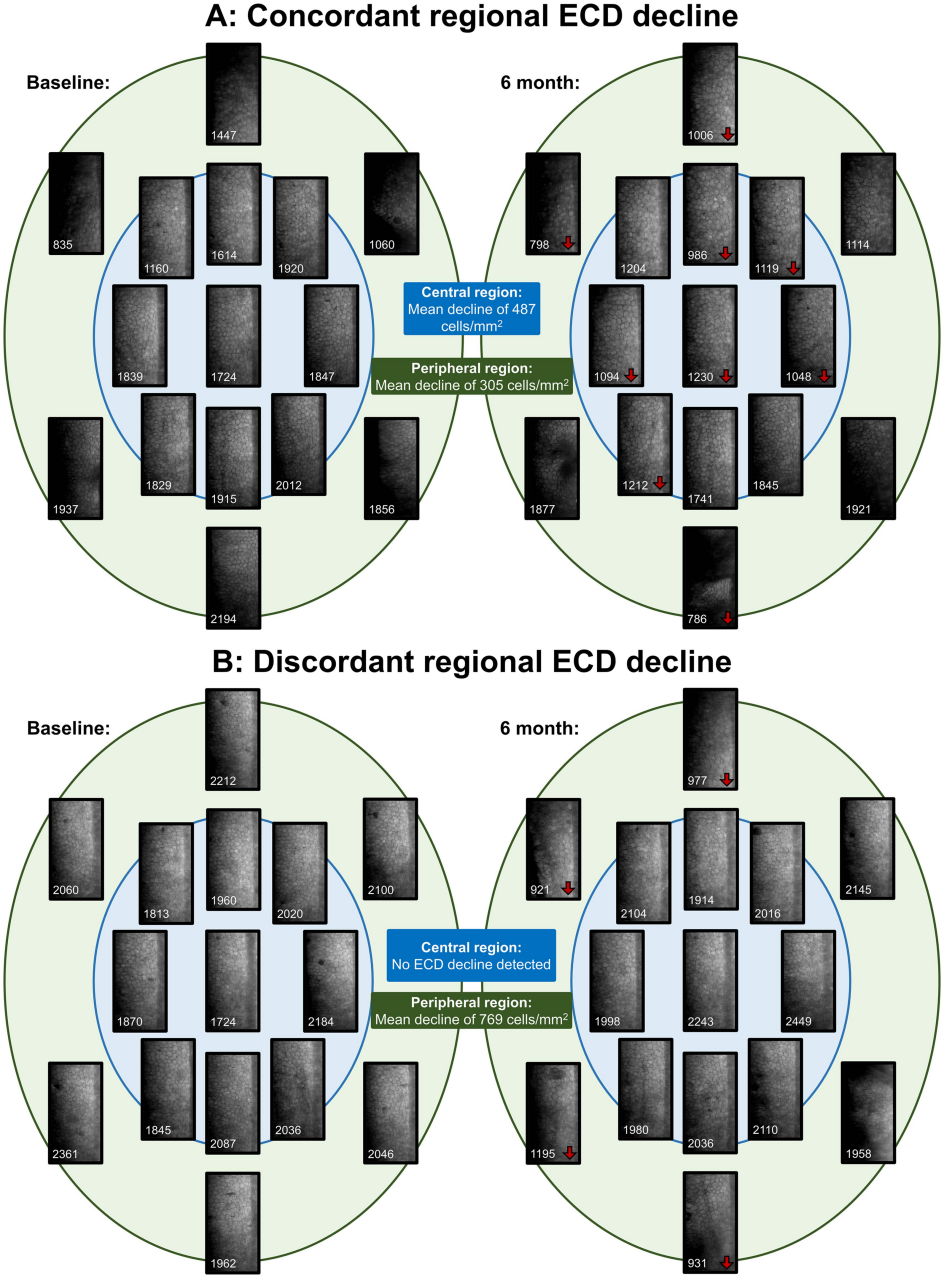
Widefield specular microscopy (WFSM) images at baseline visit and 6-month visit, highlighting endothelial cell decline (ECD) regionally. ECD for each image are marked at the bottom of the image in white, and areas that had > 10% decline in ECD were marked with red arrows next to the ECD values. **(A)** demonstrates concordant regional declines between the central and peripheral regions. This patent is a Chinese male who had DMEK at age 57 for FECD. **(B)** demonstrates discordant regional declines between the central and peripheral regions. This patent is a Chinese female who had DMEK at age 66 for FECD.

Overall, we estimated a mean annual central ECL of 5.81% (95% CIs: 3.54–8.08%) post-DMEK. Sub-analysis comparing central and peripheral ECD decline revealed that there were no significant differences between regions in mean ECD declines in unpaired (154 vs. 151 cells/mm^2^; *p* = 0.968) and paired analyses (106 vs. 151 cells/mm^2^; *p* = 0.453). Comparing FECD and PBK eyes, there were no significant differences in ECD decline at the central (143 vs. 190 cells/mm^2^; *p* = 0.596) and peripheral regions (134 vs. 213 cells/mm^2^; *p* = 0.587). DMEK donor graft ECD was detected to be inversely associated with central ECD decline in multivariable analysis (Beta = −0.591 [−1.15 - -0.034]; *p* = 0.038), adjusting for age, gender and surgical indication. There was also poor correlation between central and peripheral ECD decline (*r* = 0.245; *p* = 0.261). Two case study eyes were highlighted to illustrate regional ECD trends, with WFSM images shown at baseline and 6-month visits (Figure 3). The case study eye in Figure 3A shows concordance between the regional ECD declines, while Figure 3B demonstrates discordant regional ECD declines.

### Deep learning assessment of image quality and endothelial cell density

**Figure 4:**
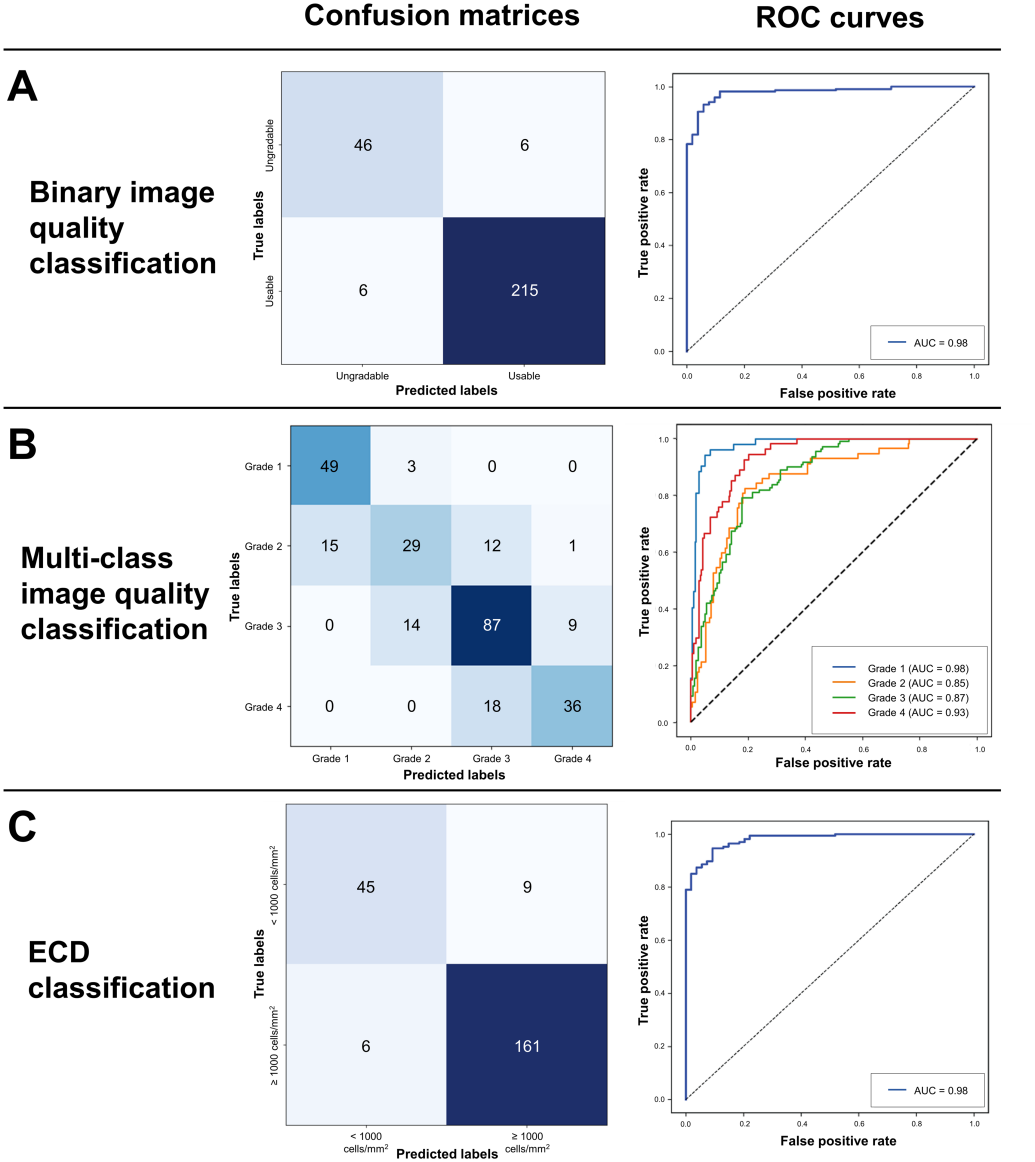
Confusion matrices and receiver operating characteristic (ROC) curves assessing the three tasks of the deep learning model for **(A)** binary image quality classification, **(B)** multi-class image quality classification and **(C)** ECD classification. Average AUCs for the tasks are 0.979, 0.907 and 0.980 respectively.

**Figure 5:**
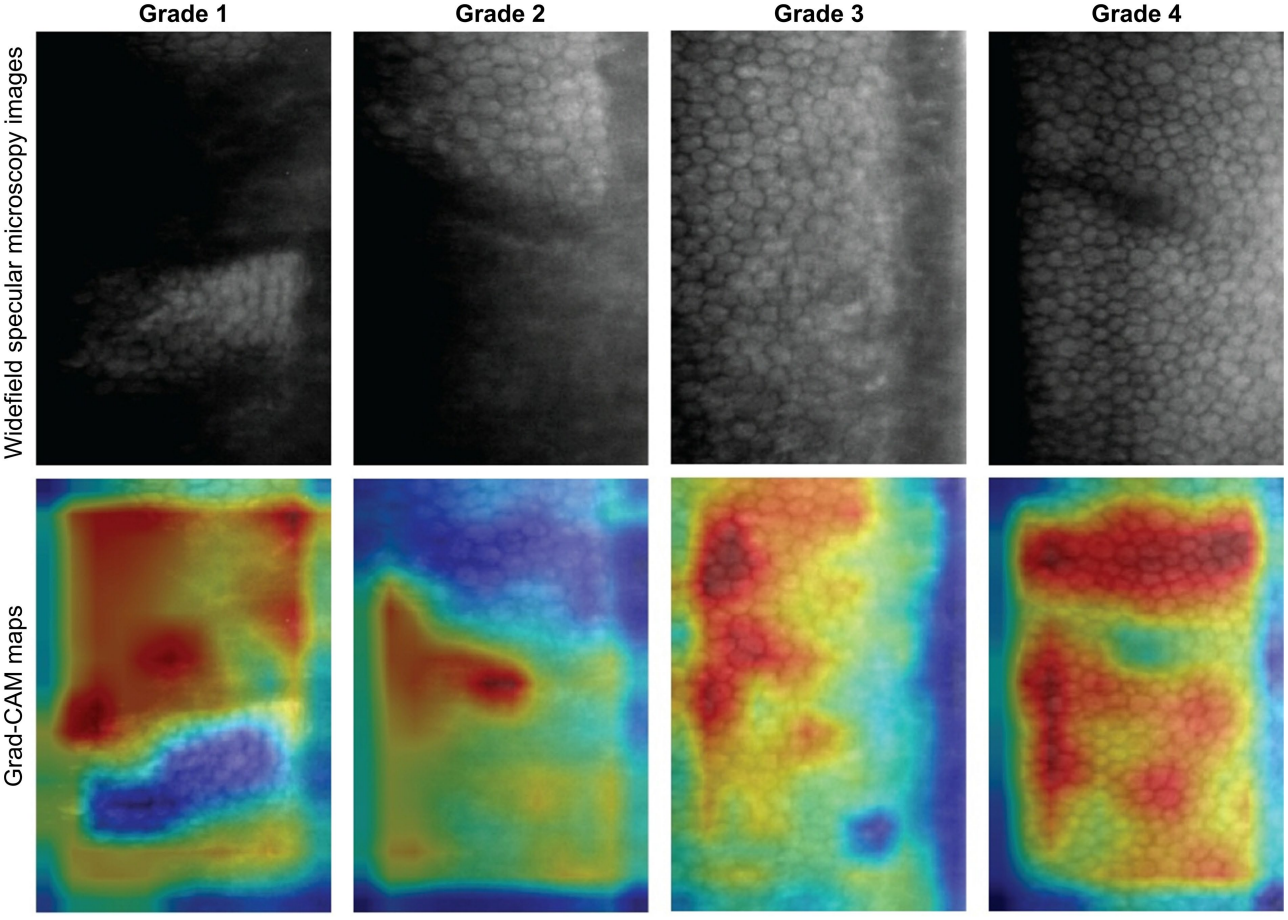
Grad-CAM visualizations for multi-class image quality grading. Representative specular microscopy images (top row) and their corresponding Grad-CAM maps (bottom row) for each quality grade. Warmer colours indicate image regions contributing most to the model’s prediction. The model focused on blurry, low-contrast, or dropout areas for lower grades (1 and 2), and on well-defined cell regions for higher grades (3 and 4). These focused features align with human expert visual grading.

We evaluated three DL classification models: (i) binary image quality grading, (ii) multi-class image quality grading, and (iii) rapid ECD screening on their respective test sets (Figure 4). The binary image quality grading model classified ungradable (grade 1) vs. usable images (grade ≥ 2) with 0.956 accuracy, 0.929 precision, recall and F1 score, and an AUC of 0.979. The multi-class image quality grading (grade 1 to 4) model obtained an overall accuracy of 0.868, 0.730 precision, 0.727 recall, F1 score of 0.723, and AUC of 0.907. The model performed best on grades 1 (AUC = 0.980) and 4 (AUC = 0.934). Misclassifications were more frequent between grades 2 and 3 due to overlapping image quality characteristics. To enhance interpretability, Grad-CAM was used to visualize image regions that contributed most to the model’s classifications. Figure 5 shows representative examples from the multi-class image quality grading task. In lower-quality images (grades 1–2), the model focused on blurred areas, signal dropout, and low contrast. For higher-quality images (grades 3–4), attention was centred on visible and well-defined cell structures. These attention patterns align with expert human grading. The ECD classification model, which aimed to detect low ECD < 1,000 cells/mm^2^, achieved 0.932 accuracy, 0.914 precision, 0.899 recall, 0.906 F1 score, and an AUC of 0.980.

## Discussion

In this ‘proof of concept’ study, we observed that WFSM was useful in detecting longitudinal declines in ECD in the central (1,709 vs. 1,555 cells/mm^2^; *p* < 0.001) and peripheral regions (1,498 vs. 1,347 cells/mm^2^; *p* = 0.016). We also demonstrated the potential for DL analysis of specular images derived from WFSM in eyes post-DMEK, achieving high diagnostic performance AUCs of 0.979 for binary image quality classification, 0.907 for multi-class image quality classification and 0.980 for detecting critical ECD threshold (< 1,000 cells/mm^2^). To our knowledge, this is the first DL approach to simultaneously assess image quality and ECD from WFSM in post-DMEK eyes, enabling early detection of CEC loss, particularly in peripheral regions where CEC migration and apoptosis typically begin—areas often overlooked by conventional central imaging. Practitioners can thus leverage this integrated assistive tool to intuitively interpret large volumes of specular images in a single session, facilitating efficient monitoring of eyes at risk of ECD loss.

While both quantitative and qualitative assessments of endothelial cell health are essential for evaluating graft integrity, image quality remains a key determinant of ECD measurement reproducibility and reliability (29). This is particularly critical for non-expert practitioners, where poor image quality, often due to corneal edema, can lead to inaccurate ECD estimations. Our DL model has effectively stratified reliable (grade ≥ 2) from unreliable (grade 1) (AUC: 0.979) as well as multi-class image quality grading (AUC: 0.907). We selected grade 2 as the cut-off for image quality as it provides adequate area (≥ 25% of image) of CEC for ECD calculation, with grade 1 images typically having no identifiable CECs in the image.

The greatest CEC loss following DMEK occurs perioperatively in the first 6 months (5, 19, 31), with reported ECL between 25 to 42% (32–34). Factors associated with perioperative ECL include graft diameter (35), type of tamponade gas (36), need for rebubbling (37), and surgical experience (38). However, beyond the initial perioperative period, the rate of ECL reduces to approximately between 3 and 6% annually after the first post-operative year (39–41). In our study of stable DMEKs, we estimated an overall average annual ECL of 5.81% which is inclusive of perioperative ECL. Long-term ECL in stable DMEK grafts is greater than physiological decline in normal corneas (42), and is associated with immune-mediated rejection (43) or non-immune mediated CEC attrition (44). However, long-term monitoring of DMEK CECs in most studies and clinical settings remain confined to the ‘central’ region of the DMEK graft. Our study underscores the importance of evaluating the corneal graft beyond the central region, as ECL can vary substantially between central and peripheral areas. Patients may exhibit early peripheral ECL that remains undetected when assessments are limited to the central cornea as illustrated in Figure 3B. This observation aligns with the study by Monnereau et al. (45), which demonstrated that early morphological changes in endothelial cells beyond the central zone may precede allograft rejection. Such peripheral changes could warrant early steroid or Rho kinase inhibitor (46) intervention to prevent irreversible graft damage associated with rejection. Future studies could correlate graft survival with regional ECL and other endothelial cell changes (45, 47).

The peripheral endothelium and the transitional zone contain endothelium progenitors (48) and influence cell migration (49–51), which are the foundation of regenerative therapies such as Rho-associated kinase inhibitors (52–54) and fibroblast growth factors (55), used alongside surgical techniques such as Descemet stripping only (DSO) (56–59) and Descemet membrane transplantation (DMT) (51, 60, 61). Furthermore, corneal regions are not perfectly symmetrical in morphology (62) and are differentially affected by pathologies (63, 64). For example, in advanced FECD, the peripheral ECD may be able to detect severity of disease better than central ECD (65). Glaucoma drainage devices have been shown to reduce ECD (66), possibly due to damage to the transition zone in the peripheral cornea (67). The above examples where monitoring of the peripheral corneal endothelium is crucial, highlight the clinical utility of WFSM. Studies have shown that healthy peripheral CECs from the host may migrate and repopulate the DMEK graft in FECD (68), whereas in PBK the peripheral endothelium is compromised and donor-to-host migration may predominate (69). However, we did not detect a difference in peripheral ECD change between FECD and PBK in our stable DMEKs. This could possibly be explored in future studies using WFSM, focusing on and investigating the early post-operative period.

**Figure 6:**
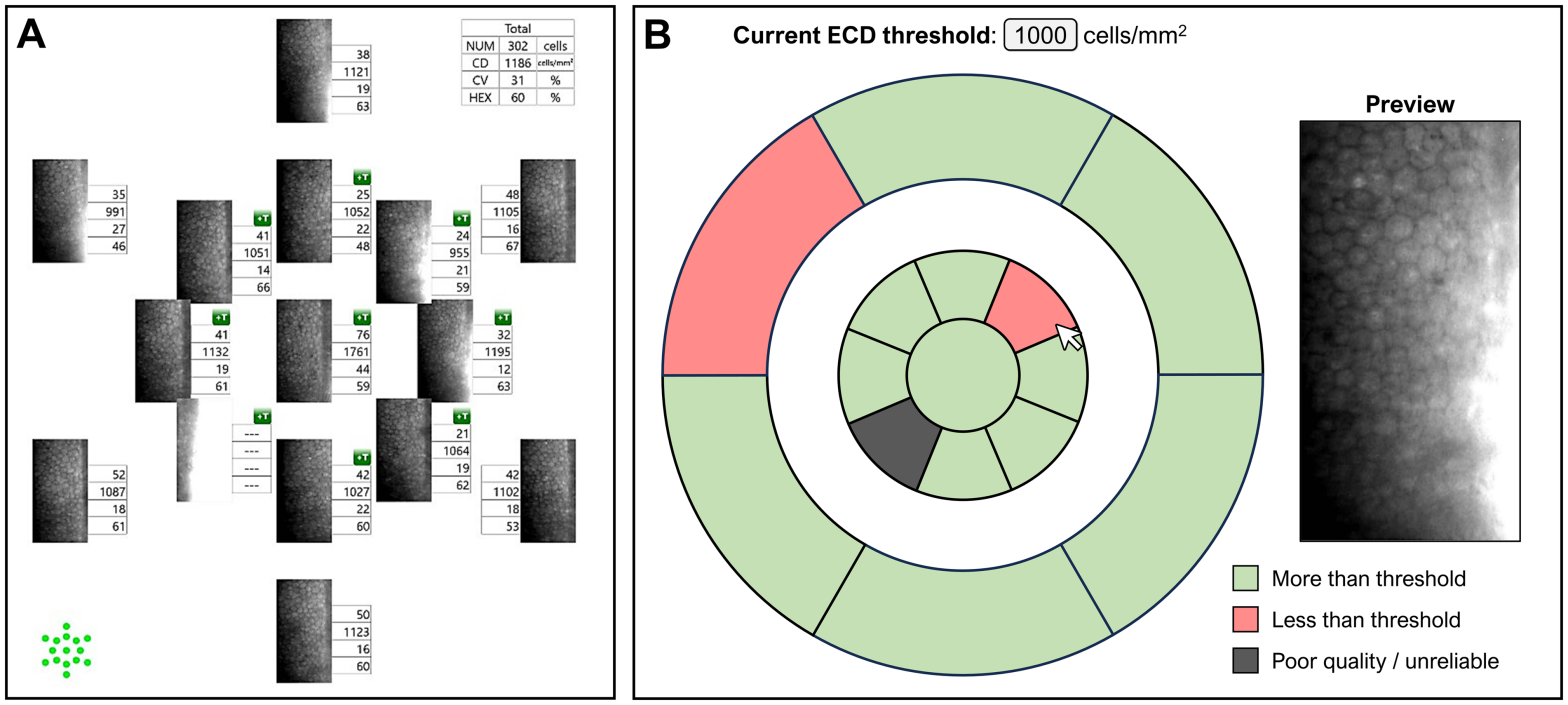
**(A)** User interface currently for Nidek WFSM scans. **(B)** Example of user interface with deep learning assisted image quality and ECD classification. The threshold can be preset by the user, and hovering the cursor over any of the areas shows a preview of that specular image.

However, while WFSM imaging rapidly produces numerous specular images across the corneal endothelium, this can be time-consuming to interpret in a busy clinical practice. A typical visit could require clinicians to interpret up to 120 images if this scan is repeated and performed in both eyes and compared over 2 visits. Thus, we developed and validated DL models that could assist clinicians in identifying high-quality WFSM images for interpretation while enabling rapid screening of ECD values based on a pre-determined threshold (set at 1,000 cells/mm^2^ in our study). In this example user interface (Figure 6), clinicians can quickly review the patient from an overview screen highlighting both image quality and low ECD detection, as opposed to scrolling through hundreds of specular images individually. This would help clinicians quickly assess the reliability of endothelial images, to then make clinical interpretations based on the best quality specular images. Future iterations of this DL model could possibly adjust for different ECD thresholds allowing greater flexibility and utility.

Our study has some limitations that should be considered when interpreting the findings. As this investigation was conducted at a single tertiary centre, it lacks multi-centre external validation, which may affect the generalisability and reliability of our results. The study population was restricted to patients with stable DMEK grafts, potentially omitting acute perioperative events that could influence endothelial cell density (ECD). However, this approach enabled us to capture a large sample of high-quality specular images, which was instrumental in developing deep learning (DL) algorithms. Importantly, it also allowed us to focus on the long-term stability of corneal endothelial cell (CEC) loss. This pilot study closely reflects our routine clinical approach to monitoring patients after DMEK surgery. Future studies with larger cohorts of DMEK patients could enable the development of algorithms capable of interpreting specular images, even in the presence of corneal edema or scarring. In this work, we introduce a novel combination of WFSM and DL models to rapidly evaluate image quality and identify low ECD below a defined threshold. This differs from most existing deep learning applications in endothelial imaging, which primarily focus on cell segmentation using convolutional neural networks such as U-Net (70–74). Automated cell segmentation has been demonstrated in specular imaging after ultra-thin DSAEK (75) and confocal microscopy after DMEK (76), highlighting the possibility of future work into DL-assisted endothelial cell segmentation of WFSM images for post-DMEK eyes. Foo et al. previously described a DL application of WFSM to identify FECD eyes with healthy peripheral endothelial reserves, achieving an AUC of 0.88 in detecting peripheral ECD < 1,000 cells/mm^2^ (77). The study presented here has additionally utilized DL to assess for image quality and reliability in DMEK eyes prior to evaluating for ECD. Overall, these studies suggest the growing potential for DL-assisted WFSM in various clinical scenarios for corneal endothelial diseases.

In conclusion, our longitudinal study on stable DMEK eyes demonstrates the potential of WFSM beyond traditional central endothelial imaging. This can be further augmented by DL models, which analysed large numbers of specular microscopy images to firstly identify scans of adequate quality and then to detect low ECD images at a pre-set threshold. This holistic and rapid assessment of could be utilized in clinical settings for monitoring of endothelial health and graft survival following DMEK.

## Data Availability

The original contributions presented in the study are included in the article/supplementary material, further inquiries can be directed to the corresponding author/s.
